# The Concentration of CMKLR1 Expression on Clinicopathological Parameters of Colorectal Cancer: A Preliminary Study

**DOI:** 10.3390/medicina57121299

**Published:** 2021-11-26

**Authors:** Paweł Kiczmer, Sylwia Mielcarska, Magdalena Chrabańska, Miriam Dawidowicz, Agnieszka Kula, Magdalena Rynkiewicz, Alicja Prawdzic Seńkowska, Dariusz Waniczek, Jerzy Piecuch, Janusz Jopek, Maciej Kajor, Elżbieta Świętochowska

**Affiliations:** 1 Department of Pathomorphology, Faculty of Medical Sciences in Zabrze, Medical University of Silesia, 40-055 Katowice, Poland; hannaonyszczuk@sum.edu.pl (M.C.); mrynkiewicz@sum.edu.pl (M.R.); 2 Department of Medical and Molecular Biology, Faculty of Medical Sciences in Zabrze, Medical University of Silesia, 41-808 Katowice, Poland; sylwiamielcarska@gmail.com (S.M.); d201069@365.sum.edu.pl (M.D.); d201070@365.sum.edu.pl (A.K.); alicja.senkowska@gmail.com (A.P.S.); eswietochowska@sum.edu.pl (E.Ś.); 3 Department of Oncological Surgery, Faculty of Medical Sciences in Zabrze, Medical University of Silesia, 40-055 Katowice, Poland; dariusz_waniczek@interia.pl; 4 Department of General and Bariatric Surgery and Emergency Medicine in Zabrze, Medical University of Silesia, 41-808 Katowice, Poland; jerzy.piecuch@wp.pl (J.P.); januszjopek@onet.eu (J.J.); 5 Department of Pathomorphology and Molecular Diagnostic, School of Medicine, Medical University of Silesia, 40-752 Katowice, Poland; katpat2@sum.edu.pl

**Keywords:** CMKLR1, MVD, colorectal cancer (CRC)

## Abstract

*Background and Objectives*: Colorectal cancer (CRC) is the second-most common cause of cancer-related deaths worldwide. Angiogenesis is crucial for cancer growth, infiltration of surrounding tissues, and metastasis and plays a key role in the pathogenesis of CRC. Chemerin/chemokine-like receptor 1 (CMKLR1) is one of the biochemical pathways involved in the regulation of angiogenesis in solid tumors. The aim of the study was to assess the CMKLR1 level in tumor and margin tissues of CRC in relation to histopathological parameters: microvessel density (MVD), budding, tumor-infiltrating lymphocytes (TILs), TNM scale, and grading. *Materials and Methods*: The study involved 43 samples of tumor and margin tissues obtained from CRC patients. To assess the concentration of CMKLR1 a commercially available enzyme-linked immunosorbent assay kit was used. For 35 cases, we performed CD34 immunostaining. The MVD, budding, and TILs were assessed using a light microscope. *Results*: The levels of CMKLR1 in both tumor and margin were negatively correlated with MVD and budding. CMKLR1 concentration in margin was higher in tissues with lymphocytic infiltration. *Conclusions*: Low vascularity and low budding are associated with higher CMKLR1 expression. CMKLR1 might play a multifunctional role in CRC pathogenesis by influencing tumor budding and peritumoral lymphocytic infiltration.

## 1. Introduction

Colorectal cancer (CRC) is the second leading cause of cancer-related death, killing annually more than 900,000 patients worldwide [1]. The key to successful treatment of CRC is early diagnosis, followed by radical surgery [2]. However, only about 39% of patients are diagnosed at an early stage of the disease, allowing for radical treatment. Once cancer has spread, the 5-year survival rate decreases rapidly, from about 90% at a localized stage to 72% at regional spread [3], and 14% if distant metastases occur [2,4]. It is therefore important to gain a better understanding of CRC biology in order to develop novel therapies and to improve patients’ survival rates.

One of the fundamentals of cancer progression is the formation of new blood vessels. Angiogenesis is a multifactorial process, which allows the tumor to enlarge, invade nearby tissues and metastasize. In addition to the well-studied proangiogenic factors, i.e., vascular endothelial growth factor (VEGF) and vascular cell adhesion molecule, recent studies mention the pathway of chemerin/chemokine-like receptor 1 (CMKLR1), as a factor involved in promoting angiogenesis [5].

CMKLR1, also known as ChemR23, is a G-protein coupled receptor, associated with the regulation of various processes in a living organism, which participates in adaptive and innate immunity [6]. Its high expression is found in plasmacytoid dendritic cells, macrophages, adipocytes, and endothelial cells [7]. It is also involved in adipogenesis and adipocytes maturation [8]. Increased activity of CMKLR1 and overexpression of its ligand, chemerin, strongly correlate with obesity, diabetes, and cardiovascular diseases, the hallmark of which is dysregulation of angiogenesis. Kaur et al. reported that CMKLR1 is expressed in endothelial cells and participates in processes of angiogenesis in cooperation with pro-inflammatory cytokines, TNF-a, IL-1b, and IL-6 [9]. The activity of CMKLR1 and chemerin is also associated with the stimulation of tumor invasion in esophageal cancer [10]. 

The main aim of our study was to determine whether the concentration of CMKLR1 in tissue is associated with microvessel density (MVD) in CRC and to assess its relationships with tumor histopathological parameters: budding and tumor-infiltrating lymphocytes assessment (TILs) [11]. MVD is an indicator of angiogenesis, reflecting tumor vascularization, which may also serve as an additional prognostic factor in various malignancies [12]. Budding is defined as an occurrence of a cluster with less than five cancer cells at the invasive front of the tumor [13]. In CRC, a high degree of budding in tumors is also suspected to be a prognostic factor associated with poor prognosis. The occurrence of TILs is related to the host immune status and has a prognostic value in CRC [11]. 

## 2. Materials and Methods

### 2.1. Study Sample

The study involved 43 samples of tumor tissue and surgical margin tissue collected during surgeries due to CRC. Patients were operated in the 1st Specialistic Hospital in Bytom and the Specialistic Hospital in Zabrze, Poland (approval of the Research Ethics Committee No. KNW/0022/KB1/42/III/14/16/18, 14.07.2020). Inclusion criteria included the confirmation of colorectal adenocarcinoma and negative surgical margin without tumor infiltration confirmed in a histological examination, patients’ age > 18 years, and signed written consent. Patients with tumors other than adenocarcinoma, presence of margin infiltration, lack of signed consent to participate in the study, age < 18 years were excluded from the study. To classify the tumor stage, the TNM staging system and grading were used. The characteristics of the study sample are presented in Table 1. 

### 2.2. Preparation of Samples for the Evaluation of CMKLR1 Level (T- Tumor Size, N- Lymphatic Nodes Involvement, M- Distant Metastases, G- Grading)

Fragments of tumor tissue and surgical margin tissue were weighted and homogenized using a PRO 200 homogenizer (PRO Scientific Inc., Oxford, CT, USA) at 10,000 rpm in nine volumes of phosphate-buffered saline (BIOMED, Lublin, Poland). The suspensions were sonicated with an ultrasonic cell disrupter (UP 100, Hilscher, Germany). Afterward, the homogenates were centrifuged at 12,000 rpm for 5 min at 4 °C. The total protein level was determined using a Universal Microplate Spectrophotometer (μQUANT, Biotek Inc., Winooski, VT, USA).

### 2.3. Evaluation of CMKLR1 Level

To assess protein levels in homogenates, an enzyme-linked immunosorbent assay (ELISA) was assayed according to the manufacturer’s procedure. CMKLR1 level was determined by the human CMKLR1 ELISA kit (Aviva Systems Biology, San Diego, CA, USA) with a sensitivity of 39 pg/mL. The absorbance of the samples was determined using a Universal Microplate Spectrophotometer (μQUANT, Biotek Inc., Winooski, VT, USA) at a wavelength of 450 nm. The obtained results were recalculated to the corresponding total protein level and presented as ng/mg of protein. 

### 2.4. Immunostaining

For 35 cases we performed CD34 immunostaining. The tissue samples were derived from formalin-fixed paraffin-embedded tissue blocks with primary CRC and tumor-free margin specimens. Then, samples underwent deparaffinization and rehydration. In the next step, antigen retrieval was performed by cooking slides in EnVision Flex Target Retrieval Solution High pH (Dako, Carpinteria, CA, USA) for 20 min at 95 °C. Prepared samples were incubated with a peroxidase-blocking reagent (Dako) and then incubated with CD34 antibody (clone: QBEnd/10 Cell Marque, Rocklin, CA, USA; incubation time: 30′; dilution: 1:150). Subsequently, they were put in EnVision FLEX HRP (Dako). Next, antigen–antibody complexes were stained using 3.3′-diaminobenzidine. Finally, tissue sections were counterstained with hematoxylin, dehydrated, and covered with coverslips for further analysis. 

### 2.5. Histological Evaluation

Histological evaluation was performed by two independent pathologists using an Olympus BX51 microscope. 

MVD was assessed on CD34 stained specimens by two independent pathologists using a light microscope in regions of the tumor invasive front counting the highest numbers of microvessels per area [14]. Initially, tumor sections were assessed at low magnification to detect tumor invasive front, then three hot spots with high vascularization were chosen. Microvessels were counted in three fields of view under 20× magnification. MVD was presented as the mean count of microvessels in the assessed fields of view; the number was adjusted by the normalization factor (1.210).

The percentage of tumor-associated lymphatic infiltration was estimated semi-quantitatively in a four-grade scale on the same H&E stained slides by the two pathologists, according to the criteria defined by Salgado et al. in breast cancer [15]. These include intratumoral lymphocytes with cell-to-cell contact between the lymphocyte and the tumor cell and stromal TILs in tumor tissue located dispersed in the stroma within the tumor cells without direct contact, including TILs at the invasive margin. According to the recommendations, stromal TILs were scored as a percentage of the stromal area alone, excluding areas occupied by carcinoma cells. Lymphatic infiltrates outside the tumor borders were not included in the evaluation. The area of lymphocyte infiltration lower than 5%, was considered TILs1; 5–25%, 25–50%, 50–75% of lymphocytes in the stroma was defined as TILs 2, TILs 3, and TILs 4, respectively. More than 75% was defined as TILs 5.

Tumor buds were estimated in one FOV at the hotspot area in the invasive front under 20× magnification. The number of buds was adjusted by the normalization factor (1.210). Budding was reported as follows: low budding: 0–4 buds; intermediate budding: 5–9 buds; high budding: > 10 buds. The mean number of buds per FOV was also used in the statistical analysis.

### 2.6. Statistical Analysis

Data distribution was assessed using the Shapiro–Wilk test. The log transformation of the levels of CMKLR1 provided a better fit to the normal distribution. Due to the Gaussian distribution of variables, data are presented as mean ± SD. To compare the tumor and margin levels, the paired Student’s *t*-test was used. Tau Kendal’s correlation coefficient was used to determine the association between the levels of the examined proteins, budding, T, and *n* parameters. *p* values < 0.05 were considered statistically significant. The statistical analysis was performed using STATISTICA 13 software (StatSoft, Tulsa, OK, USA). 

## 3. Results

We found a significant difference in the levels of CMKLR1 between the tumor and margin tissues (Table 2).

Mean MVD at invasive front in examined specimens is presented in Table 3.

Most of the examined specimens were characterized by low budding; intermediate budding was found only in fivespecimens (Table 4). The mean number of buds was 4.70 +/−4.75 (range: 0–17 buds). 

**Figure 1:**
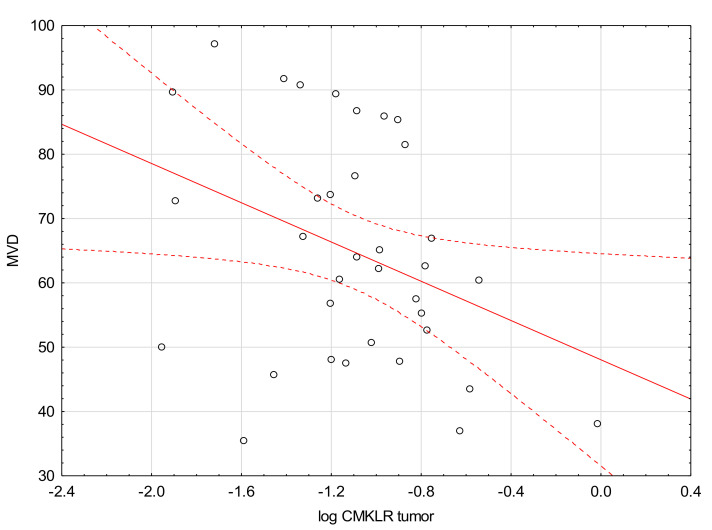
Correlation between the levels of CMKLR1 in tumor tissue and MVD (R = −0.36, *p* = 0.036).

**Figure 2:**
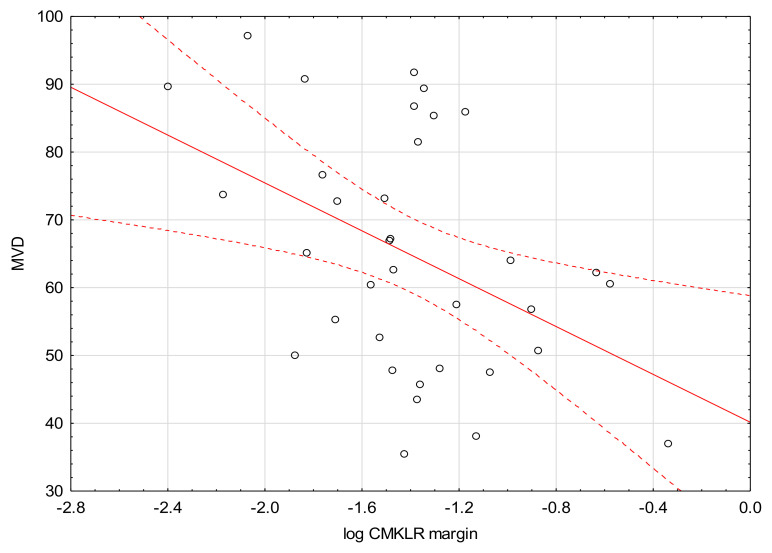
Correlation between the levels of CMKLR1 in margin tissue and MVD (R = −0.44, *p* = 0.01).

We found a negative correlation between the levels of MVD and CMKLR1 in the tumor and margin (Figure 1 and Figure 2). 

**Figure 3:**
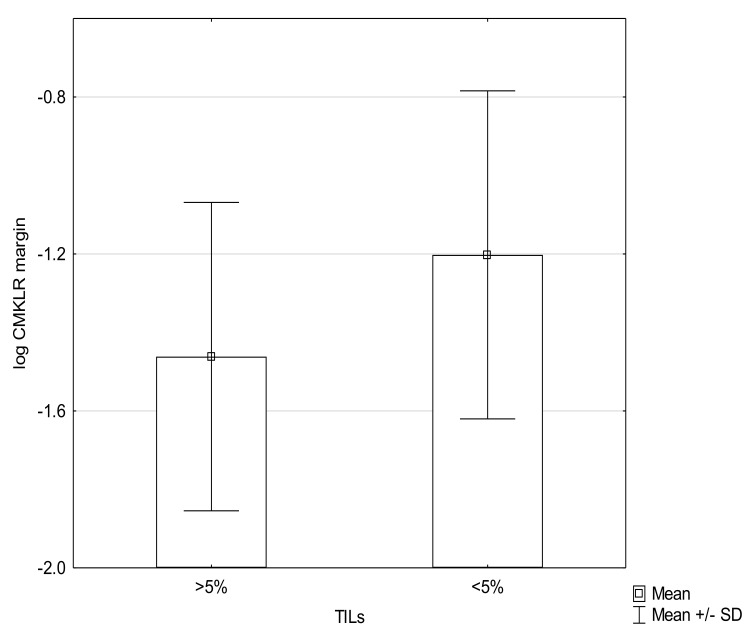
Association of CMKLR1 concentration in the margin with TILs (*p* = 0.054).

The level of CMKLR in margin tissue was higher in tumors with low immune response (TILs 1), compared with tumors with TILS >1 (mean −1.2 SD = 0.42 vs. −1.46; SD = 0.39) (Figure 3).

**Figure 4:**
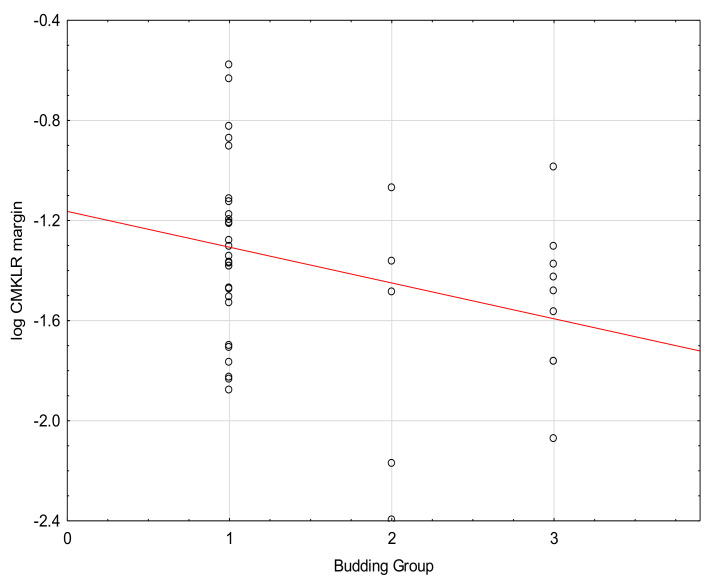
Correlation between the levels of CMKLR1 in margin tissue and Budding (Tau = −0.22, *p* = 0.05).

**Figure 5:**
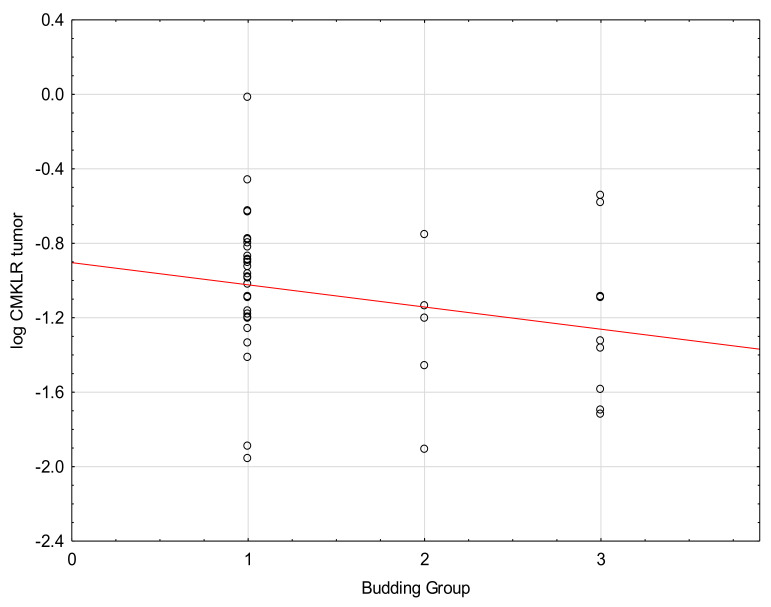
Correlation between the levels of CMKLR1 in tumor and Budding (Tau = −0.21, *p* = 0.03).

We found a significant negative correlation between budding and the levels of CMKLR in tumor and margin tissues (Figure 4 and Figure 5, Table 5. No correlations between the level of CMKLR1 and TNM or grading were found.

Neither MVD nor budding correlated significantly with TNM or grade. No significant association was observed between MVD and budding.

## 4. Discussion

Increased concentration of CMKLR1 in colorectal cancer tissue was reported in our recent study [16]. Our research and several other studies suggested that the increase in CMKLR1 concentration may be associated with the tumor progression in colorectal cancer, as well as gastric cancer [17].

Chemerin may induce pro-invasive action in gastric cancer through induction of VEGF and other factors such as matrix metalloproteinases and Il-6 [18]. Huang et al. discovered the influence of chemerin expression on the levels of VEGF via regulation of CMKLR1 and lncRNA maternally expressed gene 3 (Meg3). Both molecules are described as stimulators of angiogenesis [19]. Furthermore, CMKLR was reported to stimulate migration of endothelial cells via the Akt signaling pathway [5,9] and to upregulate matrix metalloproteinases, which, in turn, promote tumor invasion and angiogenesis [9]. Our recent studies confirmed the positive correlation between CMKLR1 and MMP-9 (Matrix metallopeptidase 9), which is involved in angiogenesis by remodeling the extracellular matrix [16]. However, in our previous study [20], we did not observe any association between CMKLR1 and VEGF A levels in either the tumor or the margin tissue of CRC. In this study, we assessed the influence of CMKLR on MVD at the invasive front of the tumor. MVD is suspected to be an independent prognostic factor in colorectal cancer [21,22]. High MVD may be associated with poorer disease-free survival in stage II CRC, yet may improve outcome among stage III cases treated with neoadjuvant therapy. Higher density of blood vessels increases the risk of vessel infiltration and cancer spread but, in turn, allows penetration of anticancer drugs through tumor tissue. However, even a dense blood vessel network may not be able to provide efficient penetration of oxygen and drugs due to the structural dysfunctions of the vessels [23].

A significant negative correlation was found between the levels of MVD and CMKLR in the tumor and margin tissues. Nevertheless, the studies reporting on the relationship between chemerin/CMKLR1expression and MVD present conflicting conclusions. Wang et al. reported that in squamous cell carcinoma of the oral tongue (SCOOT), chemerin expression correlates positively with MVD, both in the tumor and surrounding tumor-free tissue [24]. More importantly, patients with an increased level of chemerin had decreased rate of survival. On the other hand, Lin et al. revealed that in hepatocellular carcinoma (HCC) chemerin may play a completely different role as a factor that negatively regulates tumor-associated inflammation, thereby inhibiting HCC progression. According to this study, chemerin might suppress IL-6 and GM-CSF (Granulocyte-macrophage colony-stimulating factor) production, which results in impaired accumulation of myeloid-derived suppressor cells (MDSC) and formation of tumor-suppressing microenvironment with decreased angiogenesis [25]. In our study, we found higher CMKLR levels in the margin tissues of the specimens with low immunologic response assessed by TIL grade. This finding indicates a possible association between immunological processes and CMKLR1 expression in CRC.

Upregulation of CMKLR ligand, chemerin, was found in coronary arteries undergoing hypoxia [26]. In our previous study [20], we observed a positive correlation between levels of CMKLR1 and HIF-1α (hypoxia-inducible factor 1) in margin tissues of CRC. Therefore, we may suspect that CMKLR1 is upregulated in poorly vascularized tumors, probably in response to a hypoxic microenvironment. Thus, CMKLR1 expression may be more related to hypoxia than to tumor angiogenesis. However, we have to bear in mind that our study is limited by a small number of samples, and a complete assessment of CMKLR1 influence on MVD, as well as the evaluation of the role of hypoxia in the upregulation of CMKLR1 expression, requires further research. 

Assessment of budding is considered as an independent prognostic factor that may correlate with the invasion of the lymphatic and blood vessels, perineural invasion, probability of lymph nodes involvement and distant metastasis, as well as with worse prognosis and higher risk of disease recurrence [27]. Although the mechanisms underlying the tumor budding are poorly understood, epithelial to mesenchymal transition (EMT) has been reported to be one of the processes most closely associated with tumor budding. EMT allows tumor cells to lose their epithelial features (inter-cellular attachments, polarity) and gain features characteristic for mesenchymal cells, which is reflected in their increased ability to migrate and invade. During EMT, the expression of epithelial markers such as E-cadherins is decreased, while the production of mesenchymal markers, such as N-cadherins, vitronectin, fibronectin, metalloproteinases, collagen, TWIST, ZEB1/2 increases [13]. Similarly, tumor buds have been reported to express potential stem cells markers, decrease the expression of E-cadherins and increase the expression of proteins associated with tumor invasiveness such as matrix metalloproteinases, nuclear β-catenin, p16, and VEGF [13,28,29,30,31,32,33]. 

In our study, we found a negative correlation between budding and the levels of CMKLR1 in the tumor and margin tissue. The most studied process positively connected with budding is Akt pathway signaling, which induces the EMT in CRC [34]. Some studies report that hypovascular tumors are characterized by stronger budding, and budding cells express hypoxic phenotypes. However, in our study, we found no correlation between microvessel density and tumor budding. 

Nevertheless, the studies reporting on the relationship between CMKLR1 and EMT also present conflicting conclusions. Kumar et al. reported that in gastric cancer chemerin via CMKLR1 may induce changes in cellular phenotype, which are similar to EMT, and result in increased migration and invasion of tumor cells [17]. On the other hand, Kim et al. indicated that in breast cancer chemerin inhibits EMT, thereby decreasing the ability of tumor cells to migrate, invade and metastasize [35]. In addition to EMT, the tumor microenvironment, especially the presence of lymphocytic infiltration, plays an important role in tumor budding [13]. Zlobec et al. have found a negative correlation between tumor budding and lymphocytic inflammation in colorectal tumors. Additionally, this study suggests that the presence of infiltrating lymphocytes in tumors attenuates the positive relationship between the degree of budding and poor patient prognosis [36]. However, in the present study, we did not observe any correlation between TILs and budding. In esophageal cancer, the presence of tumor buds may reflect a decreased inflammatory response to tumor cells and result in worse outcomes in patients [37]. In melanoma, in vitro studies indicated that chemerin may inhibit tumor growth through alteration of immune infiltration. Expression of chemerin in melanoma tumor cells causes an increase in the number of natural killer cells and a decrease in the number of MDSC, and putative immune inhibitory plasmacytoid dendritic cells present in immune infiltration surrounding the tumor. Furthermore, the presence of CMKLR1 in melanoma cells is necessary to induce the inhibition of tumor growth and alteration in cell composition of immune infiltrates [38]. 

The tumor-infiltrating lymphocytes (TILs) were assessed in our study; however, the correlation between the TILs and concentration of CMKLR1 in tumor tissue was insignificant. The levels of CMKLR in the margin tissue were lower in tumors with lymphocytic infiltration, defined as TILs > 1. We may suspect that chemerin via CMKLR1 plays a role in the modification of the immune infiltration by decreasing the number of TILs. Some studies report that activation of CMKLR1 by chemerin may exert antitumoral effects via recruiting antitumoral immune cells such as NK cells and suppressing M2 macrophages involved in tumor invasion [39]. Recent studies suggest that chemerin/CMKLR may suppress tumor growth via stimulating T-cell-associated cytotoxicity via PTEN (phosphatase and tensin homolog deleted on chromosome ten) and PD-L1 (Programmed death-ligand 1) [40]. Due to the fact that the ELISA technique provides only quantitative measurement of CMKLR1 concentration in tissue homogenates obtained from tumor and margin tissue, further research with the use of IHC (Immunohistochemical) staining is needed to determine the localization of CMKLR1 expression, both in tumor and tumor-infiltrating lymphocytes (TILs).

Based on our findings, we may conclude that low vascularity, as well as low budding, are associated with higher CMKLR1 expression. Further studies should be performed to precisely determine the role of CMKLR in EMT and angiogenesis. Based on previous studies, we may suspect that upregulation of CMKLR in tumors may be associated with poor vasculature and hypoxia [20]. To the best of our knowledge, tumor budding is related to poorly developed vasculature. This association was not confirmed in our study; thus, there may be other factors determining the tumor behavior. Although we did not find significant correlations between CMKLR1 concentration and TILs, we observed a higher level of CMKLR1 in the margin tissue in tumors with a low immune response (TILs 1). This finding suggests that chemerin and CMKLR1 may contribute to tumor progression and also have a role in the antitumoral immune response. CMKLR activity may be involved in both cancer progression and immunologic response against the tumor. Thus, investigating the role of this receptor in colorectal cancer pathogenesis may contribute to finding a new aim for target therapy of colorectal cancer.

The results of our study do not show any correlations between the levels of CMKLR and clinicopathological parameters, such as TNM or grading. Lack of associations may be due to the small and homogeneous study group. It is thus necessary to perform further research involving a larger sample size. 

## 5. Conclusions

In conclusion, the CMKLR receptor may play a multifunctional role in CRC pathogenesis, which may result in turn in influencing tumor budding and peritumoral lymphocytic infiltration. CMKLR1 upregulation in CRC may be associated with a hypoxic environment and low vascularity.

## Figures and Tables

**Table 1:** Patient characteristics.

		*n*	%
T	1	0	0.00
	2	14	32.56
	3	20	46.51
	4	9	20.93
N	0	26	60.47
	1	11	25.58
	2	6	13.95
M	0	35	81.40
	1	8	18.60
G	1	3	6.98
	2	38	88.37
	3	2	4.65
Stage	I	12	27.91
	II	11	25.58
	III	12	27.91
	IV	8	18.60

**Table 2:** CMKLR1 (chemerin/chemokine-like receptor 1) concentrations in tumor and margin.

CMKLR1 Concentration	Mean	SD	*p*
Log CMKLR1 tumor	−1.09	0.40	<0.0001
Log CMKLR1 margin	−1.38	0.41	

**Table 3:** Mean MVD (microvessel density) at invasive front in the examined specimens.

Histological Parameter					
	*n*	Mean	Min	Max	SD
MVD	35	64.82	35.46	97.10	17.58

**Table 4:** Budding and TILs (Tumor infiltrating lymphocytes) assessment in examined specimens.

		*n*	%
Budding	0–4	29	67.44
	5–9	5	11.63
	>10	9	20.93
TILs	0–5%	14	32.56
	6–25%	17	39.53
	26–50%	8	18.60
	51–75%	3	6.98
	>75%	1	2.33

**Table 5:** Correlations between CMKLR1 concentration, budding and TILs (Tau Kendall’s correlation).

CMKLR1 Concentration	*n* = 43		
		Tau	*p*
Log CMKLR1 tumor	TILS	0.18	0.09
	Budding	−0.21	0.05
Log CMKLR1 margin	TILS	−0.07	0.51
	Budding	−0.22	0.03
